# Visioning for secondary palliative care service hubs in rural communities: a qualitative case study from British Columbia's interior

**DOI:** 10.1186/1472-684X-8-15

**Published:** 2009-10-09

**Authors:** Valorie A Crooks, Heather Castleden, Nadine Schuurman, Neil Hanlon

**Affiliations:** 1 Department of Geography, Simon Fraser University, 8888 University Drive Burnaby, British Columbia, V5A 1S6, Canada; 2 School of Resource & Environmental Studies, Dalhousie University, 6100 University Avenue, Halifax, Nova Scotia, B3H 3J5, Canada; 3 Geography Program, University of Northern British Columbia, 3333 University Way, Prince George, British Columbia, V2N 4Z9, Canada

## Abstract

**Background:**

As the populations of many developed nations continue to age at rapid rates it is becoming increasingly important to enhance palliative care service delivery in order to meet anticipated demand. Rural areas face a number of challenges in doing this, and thus dedicated attention must be given to determining how to best enhance service delivery in ways that are sensitive to their particular needs. The purposes of this article are to determine the vision for establishing secondary palliative care service hubs (SPCH) in rural communities through undertaking a case study, and to ascertain the criteria that need to be considered when siting such hubs.

**Methods:**

A rural region of British Columbia, Canada was selected for primary data collection, which took place over a five-month period in 2008. Formal and informal palliative care providers (*n *= 31) were interviewed. A purposeful recruitment strategy was used to maximize occupational and practice diversity. Interviews were conducted by phone using a semi-structured guide. Interviews were audio recorded and transcribed verbatim. Data were managed using NVivo8™ software and analyzed thematically, using investigator triangulation to strengthen interpretation.

**Results:**

Four themes emerged from the dataset: (1) main SPCH features; (2) determining a location; (3) value-added outcomes; and (4) key considerations. It was found that participants generally supported implementing a SPCH in the rural region of focus. Several consistent messages emerged, including that: (1) SPCHs must create opportunities for two-way information exchange between specialists and generalists and communities; (2) SPCHs should diffuse information and ideas throughout the region, thus serving as a locus for education and a means of enhancing training opportunities; and (3) hubs need not be physical sites in the community (e.g., an office in a hospice or hospital), but may be virtual or take other forms based upon local needs.

**Conclusion:**

Visioning innovation in the provision of palliative care service in rural communities can be enhanced by consultation with local providers. Interviews are a means of determining local concerns and priorities. There was widespread support for SPCH coupled with some uncertainty about means of implementation.

## Background

Palliative care comes in many forms, but in general this term refers to support provided to maintain quality of life for individuals living with chronic conditions, and the facilitation of quality of death for persons in the end stages of disease or infirmary [1]. Rural communities face particular challenges in providing diverse and specialized forms of palliation, along with other health services. For example, sparse populations in rural areas make it difficult to attract a broad array of primary care providers and specialists, thus narrowing the range of palliative care expertise available to residents [2,3]. This service issue is particularly acute in many developed nations that are home to rapidly aging populations and thus have a pressing need to enhance palliative care service provision [4].

Broadly speaking, the literature on rural palliative care services tells us that: average final hospital stays are longer in rural hospitals than in urban ones [3], rural residents are more likely to die out of hospital [5], and models of care delivery designed and tested in urban centres may not work in desired ways in rural communities [3]. Meanwhile, increasing numbers of people are moving to such communities upon retirement while other still are 'aging-in-place' [6,7], both of which are further amplifying the demand for palliative care services in rural areas [8]. Dying individuals' frequent desires to remain in their homes or home communities for as long as possible, including in rural areas, further necessitates attentiveness to this important health service issue [3,9].

Given the circumstances outlined above, novel approaches are thus needed to meet the unique health service demands of rural communities [10,11]. More specifically, innovation is required in order to address the health service need to provide palliative care to rural residents [2,3,12]. There have been minimal attempts to address this need from the research community; however, an important exception has been Kelley's [13] work on building community capacity for palliative care in rural Canadian communities. In the present article we advance the vision of a service delivery model that may assist with meeting this health service need: namely that of the secondary palliative care service hub (SPCH). Primary hubs are understood to be larger centres (e.g., regional hospitals) that offer specialized palliative care [14], which is expert care delivered by a trained multi-professional team [15]. Working from an acknowledgement that it is typically not possible to offer specialized services in smaller rural centres, SPCHs are *carefully identified *rural communities that are most in need of having, and are thus most likely to benefit from, enhanced links with primary hub sites (i.e., larger hospitals). This characterization is intentionally open as this service approach is refined in the present article through undertaking a case study in a particular rural region of the Canadian province of British Columbia (BC). We consider the SPCH model to be advantageous because as primary care providers undertake most of the palliative care service provision in rural areas [16], creating links between primary hub sites where specialists practice and SPCH-hosting communities may enhance on-the-ground practice.

It was mentioned above that SPCH hosting communities are intended to be thoughtfully identified based on factors such as anticipated service need [17]. Using the case study presented herein, we aim to further identify decision-making factors that should be taken into consideration when siting SPCHs. This serves as one of the purposes of the present article, which are to: (1) refine the vision of the SPCH through determining how formal and informal palliative care providers in a rural BC region determined to be lacking in palliative care services respond to this service approach, including whether and how they would apply it locally; and (2) determine those criteria that should be considered when siting a SPCH from the perspectives of these same providers. Such a focus provides information necessary for evidence-based health service allocation decision-making, which is viewed as far more desirable than haphazardly- or politically-motivated decision-making [18]. In the remainder of this article we further explore the idea of SPCHs as a service delivery model for enhancing palliative care services in rural communities by drawing on the findings of 31 semi-structured interviews conducted with formal and informal palliative care providers in the case study region.

## Methods

The goal of the larger study to which this analysis contributes was to examine palliative care service provision in a rural BC region determined to be lacking in palliative care services in order to test responsiveness to the SPCH approach and to determine localized barriers and facilitators to existing service delivery. In order to achieve this, phone interviews were undertaken with formal (e.g., family doctors, nurses, health service administrators) and informal (e.g., family caregivers, volunteers, pastors) palliative care providers in three communities in the case study region.

### Case Study Site Selection & Overview

**Figure 1:**
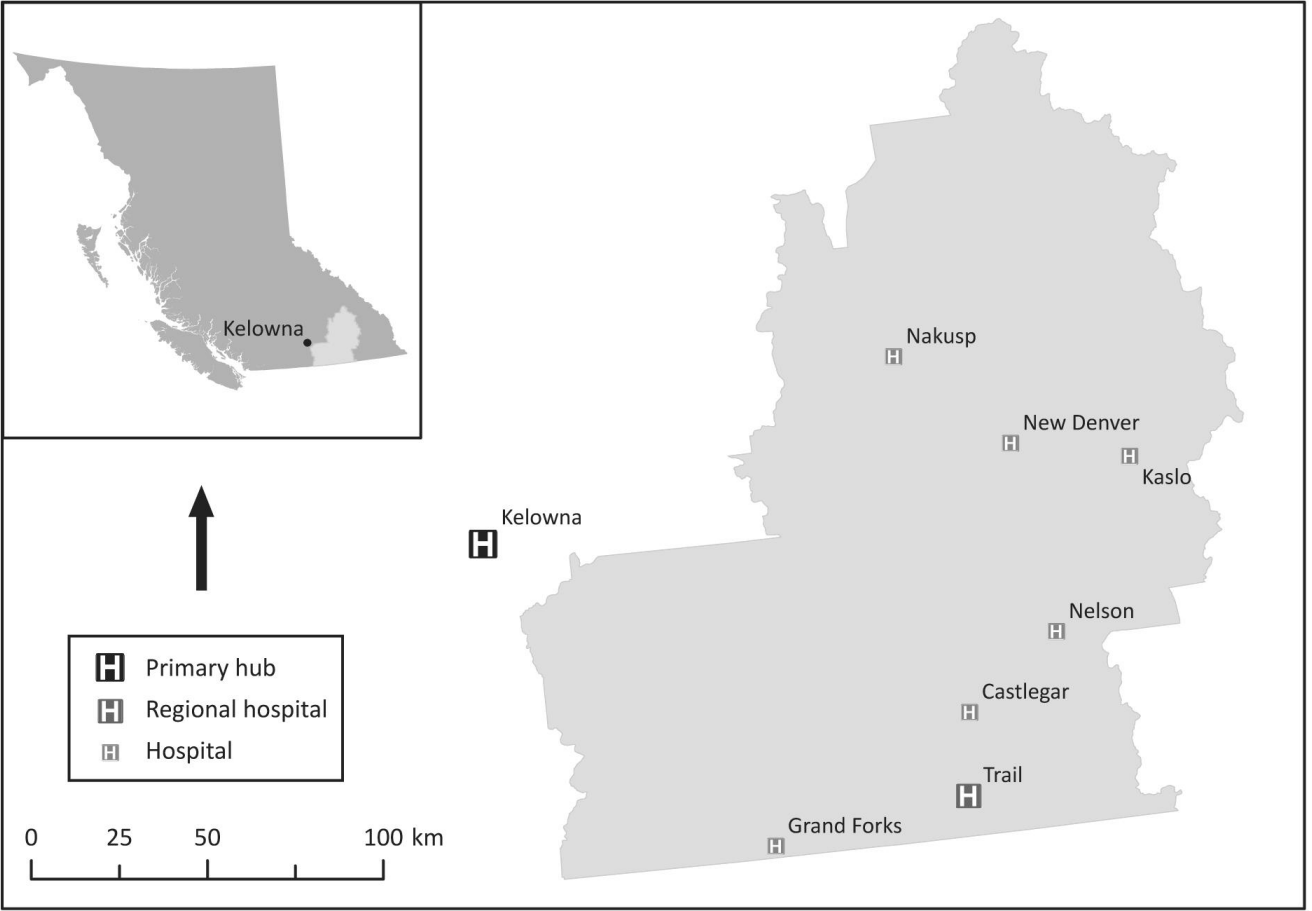
**British Columbia's West Kootenay-Boundary Region**. This map shows the location of the case study region within the province of BC.

The area of focus, the West Kootenay-Boundary (WKB) region, was identified for this case study through a series of spatial analyses conducted, first, to identify palliative care-poor regions in BC [14] and, second, to apply a model developed to identify specific communities most suitable to enhance their palliative care provision by assessing population (i.e., number of people within a one hour drive), isolation (i.e., distance from existing primary hubs), and vulnerability (i.e., number of people over the age of 65) [17]. The WKB region is located in BC's interior and is characterized by dispersed and, for the most part, lightly populated small cities and towns spread throughout a mountainous area (see Figure 1). Based on our initial spatial analyses, the communities of Nelson, Castlegar, and Trail in the WKB region were identified to potentially be the most suitable SPCHs sites throughout the entirety of rural BC, and thus were focused upon for primary data collection. Between these communities there are several health care clinics, three small hospitals, approximately eight beds designated for palliative care in extended care facilities and hospitals, no freestanding hospices, and just over 35,000 residents.

### Recruitment

A purposeful strategy was used when recruiting phone interview participants from the three communities in order to maximize diversity in terms of both occupation and practice location. First, an initial group of potential participants was contacted about the study using investigators' networks. Following this, calls for participants were placed on electronic listservs run by key associations. We further recruited through reviewing employee listings for local health services and the regional health authority, through snowball sampling from existing participants (i.e., asking existing participants to suggest other potential participants), and through targeted internet searches.

A total of 40 people were invited to participate in an interview, 31 of whom ultimately took part: seven nurses, six health service administrators, five hospice/palliative care volunteers, two family doctors, two pastors, two hospice society workers, two allied health care professionals, one family caregiver, and four others whose jobs involved palliative care provision. They identified their main community of practice to be: Nelson (*n *= 5), Trail (*n *= 11), and Castlegar (*n *= 13); though many provided services in multiple communities. The remaining two participants were based in rural northern BC and had familiarity with the state of rural palliative care provision throughout the province. It was determined that their input would provide an important comparative perspective, namely between the interior and the north, and so they were interviewed. Interestingly, their responses were complementary to those offered by the respondents from the three case study communities and so were integrated into, rather than compared to, the dataset.

### Data Collection

Participants received detailed information about the study along with a consent form that they signed and returned prior to the interview. This procedure for obtaining consent and all other aspects of the study were reviewed and approved by the research ethics offices at Simon Fraser University and the Interior Health Authority. Phone interviews were conducted by a single interviewer (the second author) in order to enhance consistency and lasted on average 1.5-2 hours. They were conducted over a five month period in 2008. Participants were asked about: experience with palliative care provision (1 question); community descriptions (3 questions); community health and health care priorities and challenges (2 questions); community need for palliative care and existing availability (10 questions); and the SPCH approach (6 questions). With the exception of a series of 21 short Likert scale questions asked at the end of the interview, which probed a range of factors that were thought to potentially assist with contextualizing participants' discussions of the SPCH approach by differentiating levels of importance, the questions posed were open-ended.

It was recognized that participants might not be familiar with a hub approach to service provision in advance of the interview. To address this, a preface to the SPCH questions was offered, whereby participants were told:

One approach for delivering health services is to create hubs. Hubs are places where services and service providers are clustered. In BC we would think of cities like Vancouver and Victoria [main urban centres] as being major hubs. In your region we know that Kamloops, Vernon, Kelowna and Penticton [main regional cities] are hubs of sorts for palliative care, as they have specialized palliative care services on site. In this study we are exploring the idea of 'secondary hubs'. These would be rural communities that draw on the expertise and knowledge available in the hubs through, for example, videoconferencing and developing mentorship relationships, in order to deliver more localized palliative care.

This excerpt comes directly from the interview guide. The preface was intended to offer some guidance on the concept without being restrictive. It was given after participants were asked the broad question: "What do you think is the best way to provide palliative care services in your community? How about in your region? And how about for the province as a whole?" Asking this question before discussing the SPCH approach was done purposely so that participants could share their general ideas without being limited by thinking about a hub design. In fact, ideas raised in response to this question were then typically related to the SPCH approach by interviewees.

### Data Analysis

All interviews were digitally recorded and transcribed verbatim. Following transcription, the documents were entered into NVivo8™, a qualitative data management program, and thematic coding ensued. A meeting was held among the investigators in order to identify issues emergent from the dataset following a detailed transcript review as a way of guiding the coding. Five major analytic issues were identified and confirmed by three investigators (namely: Aboriginal palliative care in the region, the local politics of palliative care, health service and administration issues, visioning for SPCHs, and the social and physical place of palliative care in the WKB), one of which forms the basis of this article. Organizational codes relevant to the analytic issues were then determined and applied to the dataset. Upon completion of the coding, a thematic analysis was undertaken. This resulted in the identification of four themes - discussed in detail below - that crosscut the analytic issue of visioning for SPCHs. Our analytic approach is consistent with the adopted technique, whereby thematic analysis involves categorizing coded data based on patterns that are evident within the dataset and comparing such themes to the study purpose(s) and existing literature [19]. Interpretation of the themes was confirmed across investigators in order to enhance the rigour of the analytic process. More specifically, our use of investigator triangulation in order to independently and then collaboratively identify emergent issues, generate analytic themes, and interpret themes through reviewing extracted data was done to strengthen the credibility and integrity of the findings shared herein [20].

## Results

From the interviews it was clear that participants supported the idea of enhancing palliative care service provision in the WKB region, indicating that there is great need to do so. Their answers to the wide-ranging Likert scale questions, recorded in Additional File 1: Table S1, revealed that they had strong responses to a number of issues related to palliative care provision locally and in the region, whether regarding the travel required to access services or having available the supports needed to assist people with dying at home (e.g., home care, family caregiver supports). When presented with the brief description of the SPCH shared above, all participants responded to our questions about this approach, and most suggested that such an approach would likely assist with addressing service need in the region. Using their input, it is possible further to refine the vision for the secondary hub in terms of its actual features. Four major themes were identified related to the analytic issue of visioning for SPCHs, which we expand upon in this section: (1) main SPCH features; (2) factors to consider when siting a hub; (3) value-added outcomes; and (4) key considerations in operationalizing the SPCH model. In the remainder of this section we elaborate on the findings central to these four themes in order to refine the SPCH vision.

### Main Features

While participants grappled with the idea of what a SPCH could be at the local scale (e.g., a physical entity such as a free-standing hospice, a centre of operations for housing specialists, a virtual network), they emphasized that a secondary hub needed to create opportunities for two-way information exchange between specialists in the region and local generalists. It was suggested that this could happen in a number of ways, such as through: establishing mentorship relationships between primary and secondary hub providers; and enhancing dedicated channels of communication between the primary and secondary hubs by regularly employing tele- and video-conferencing for professional networking, training, and patient management. *"Any support with education is always really welcome, whether it's funding or visiting instructors, specialists... We're always sending people away to take the training... [The] introduction of... [and] growing use of tele-health will be great." *Importantly, participants alluded to the fact that while a SPCH would provide opportunities for information to trickle down from specialists affiliated with the primary hub, there was also opportunity for deeply contextualized local information about, for example, a client's health history or local provider informational needs to trickle up. This information would not only enhance the quality and precision of expert advice coming from the primary hub, but it would also serve to enhance the professional knowledge of providers practicing in both locations.

Participants saw tremendous value in enhancing local and regional palliative care educational and training opportunities through the SPCH, particularly those that could be run jointly by providers in primary and secondary hub sites (see responses to questions 20 and 21 in Additional File 1: Table S1). *"I think we really need to support the wonderful people that are out there in all these communities...who are delivering really good care." *It was noted that one way to support providers was through education and training. It was further suggested that the SPCH could create an information clearinghouse for the region's various formal and informal palliative care providers, in terms of gaining access to both a depth and breadth of knowledge regarding the multiple dimensions of palliative care. Access to information held within the SPCH was not to be limited to formal service providers alone. For community members, having access to needed information was thought to have the potential to alleviate fears related to providing in-home care for a dying individual. As shown in Additional File 1: Table S1 (see question 19), the overwhelming majority of participants also thought it was either very or critically important for community members in general to become informed about what palliative care services were available. Circulating such details could certainly be part of the information-disseminating role of a region's SPCH. For professionals and volunteers, sharing information among provider groups was thought to have the potential to break down professional 'turf wars' and create an environment of collaboration rather than competition both within and between primary and secondary hubs. Finally, making education and training an expressed role of the exchange that is to happen between primary and secondary hubs was thought to allow resources to flow bi-directionally.

### Determining a Location

At the local scale, participants identified a consistent set of factors that needed to be considered when siting a SPCH: the availability of transportation in order to ensure access to services; the existing level of community awareness concerning palliative care; the existence of services and providers in the community and region with established palliative care expertise; and the community's proximity to a full-service tertiary hospital. Transportation and travel were the most commonly discussed factors, particularly with regard to the WKB's mountainous nature and bad winter weather. Concern regarding transportation was demonstrated in participants' responses to the Likert scale questions (see questions 9 and 10 on Additional File 1: Table S1), whereby their answers suggest more concern for travelling across the region (e.g., between communities) in bad weather than within their own communities. However, they were quick to acknowledge that in rural communities travel to access health services was routine: *"...the big problem would be transportation, and that's always an issue here." *Participants also pointed out that the ability to increase local palliative care awareness would likely be a useful criterion for determining an appropriate hub location, the reason given that such awareness could be used as leverage for additional resources: "*Raise the awareness of palliative care, and in raising that awareness, increase the availability." *Finally, while the WKB region has been distinguished as palliative care-poor, individuals with various types of local palliative care expertise do practice there, and participants suggested that a SPCH built around the location of that expertise made sense.

At the regional scale, participants' vision for the location of a SPCH included the need to have a strong connection to the region's primary palliative care hub (in this case Kelowna - see Figure 1), so as to enhance the *"...consistency of shared information." *This connection was intended to support two-way communication between professionals, prevent unnecessary duplication of services, and open a direct pathway for the potential utilization of specialized services by residents of smaller rural communities. To varying degrees, physical geography (e.g., can people literally get to and from the community with ease despite the mountainous landscape?), road networks (e.g., are their main roads connecting the community with others?), size of population (e.g., are there enough people in the region to sustain use of the hub?), professional capacity (e.g., is there the necessary clinical expertise in the region to warrant a hub being sited there?), and communities' political will (e.g., do health service enhancements typically go to a particular community in the region?) all factored into participants' criteria for determining location. Consequently, they largely advocated for physical centrality within the WKB in a site that supported the region's main transportation routes (road and air) both to the primary hub and the surrounding communities that also housed a comparatively large population with access to financial and expert human resources. Finally, the future location of a SPCH proximal to or on-site at the regional hospital in Trail (see Figure 1) was recognized as a plausible decision-making measure, though not necessarily a desirable one.

### Value-Added Outcomes

There was strong agreement that a SPCH would serve to enhance the quality of both local and regional palliative care services, this being an important outcome for clients and families. *" [A SPCH] would raise the bar for palliative care, which is going to benefit the patients and their families, because they're going to be getting a more rounded, kind of boutique, service compared to what's there right now." *Participants rationalized that the two-way information exchange, educational opportunities, and training opportunities identified as central to the SPCH approach would primarily contribute to this outcome. *"You increase your expertise in that field because then the people there, who are interested in it, could do things that provide a better service." *Further, by creating enhanced training opportunities, the region's formal and informal providers would be able to develop confidence not only in their own skills, but also in the skills of their colleagues across professions and communities. In so doing, they would also be able to systematically identify where gaps in knowledge existed and subsequently work towards filling them using a strategic approach, thus further enhancing care quality outputs.

Participants were asked to describe the profile of palliative care in the WKB region in relation to other health and health care issues. For the most part, they viewed palliative care as being neither on the public 'radar screen' nor the political agenda. One participant described the fit of palliative care into the community's priorities in the following way: *"Everybody gives lip service to it. But the reality is that it's only, you know, it's lip service." *However, participants viewed a SPCH as having the potential to: raise awareness about palliative care, both locally and regionally; and serve as a catalyst for building momentum, in terms of bringing further palliative care resources, such as a free-standing hospice, to rural communities in the region (see also Additional File 1: Table S1, question 1). Awareness and visibility were thus viewed as important outcomes of establishing a SPCH.

### Key Considerations

Participants were clear that if a SPCH were to be sited in their region, a key consideration would be to reflect carefully on who should be involved in the decision-making process regarding its location and overall form. A number of different stakeholders were easily identified by participants, including: governmental health agencies, family doctors, home care nurses, nurses working in clinical settings, spiritual care providers, alternative and allied health care providers, representatives from local volunteer hospice societies, and informal family caregivers. Each of these stakeholder groups was thought to have vested interests in terms of projecting and implementing their vision for a SPCH. It was also acknowledged that how decisions regarding the actual form of the SPCH (e.g., virtual network for mentorship and training versus a new physical site [e.g. hospice] in the hub community) were to be made would require careful attention to ensure that a lasting commitment is sustained. *"I really think...if management is not committed [then] once again, people just sort of feel...nothing's ever going to change. But I think if you have people in the same room, talking about the same issues and so that everybody has an understanding...and are...willing to sort of look outside the box." *It is clear from participants' comments that community, professional, and governmental politics would undoubtedly all need to play a major role in the decision-making processes.

Three additional key considerations to be made in determining the location and overall form of a SPCH were raised by participants. These considerations pertained to service providers in the host community, whereby it was noted that there would be: a continued need for highly committed palliative care providers; a pressing need to address any existing issues of understaffing in palliative care; and a need to address professional 'turf wars' amongst providers within and across communities in the region. It was made clear that existing informal and formal palliative care providers in the WKB region were incredibly dedicated to providing excellence and raising awareness. At the same time, participants were sensitive to the fact that their colleagues' unwavering passion also ran the risk of leading to burn out. There was, thus, a need to share the work through increased staffing efforts, which is an issue that participants thought a SPCH could champion. A hub was also thought to have the potential to attract highly qualified personnel to rural communities and to assist with reducing professional isolation and enhancing local and regional mentorship. It was clear from the participants' vision that a SPCH was equally intended to bring the various palliative care provider groups together in the spirit of knowledge mobilization and information sharing within and across rural communities. *"The biggest benefit [of] the hub [is] having that process for staff...whether it be physicians, pharmacists or other...residential care facilities who have palliating residents here, it's a resource for everybody...a known resource that would be [seen as] the place to go...setting the bar as far as best practice." *It was thought that this may, in turn, lessen internal political strife while enhancing interprofessional collaboration.

## Discussion

**Figure 2:**
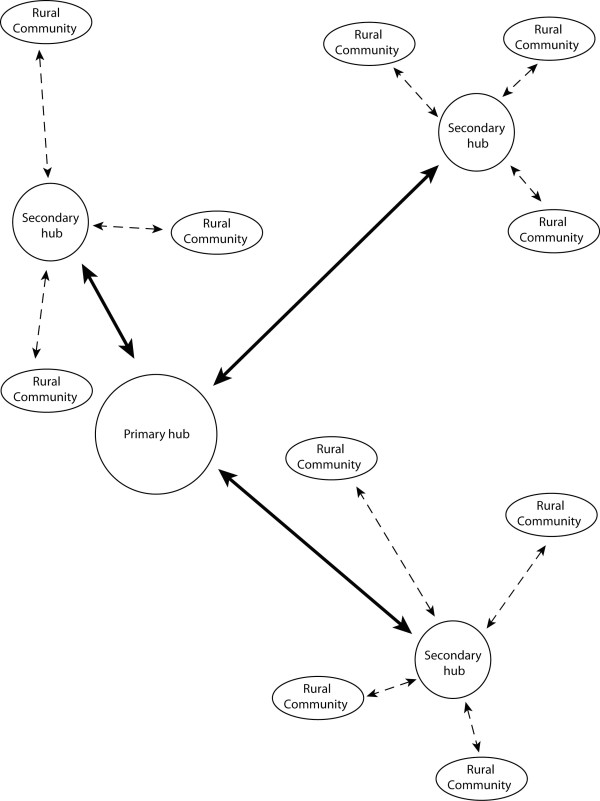
**Visualizing the Secondary Palliative Care Hub**. This figure provides a visual illustration of several of the features of the SPCH that participants identified as desirable. The primary hub (e.g., a tertiary hospital with full palliative care services) has: specialist knowledge; existing training opportunities; diagnostic tools and expertise; and multi-professional team practice. The secondary hub (e.g., a rural community that has primary care practitioners, other formal and informal providers, and possibly even a local hospice) has: generalist knowledge; a desire to build capacity; places where educational and training events can be hosted from; and a vision to enhance care provision. Resources shared between the primary and secondary hubs can include: patient information; mentorship; teleconferencing; joint educational and training opportunities; and diagnostic and symptom management assistance. Noteworthy is that exchange is not limited to the primary and secondary hubs; other rural communities in the region can benefit from the existence of a secondary hub in the area through site visits and information and knowledge exchange.

It was stated at the outset that the purposes of this article have been to use the case study interviews to: (1) refine the vision of the SPCH; and (2) determine those criteria that should be considered when siting a SPCH. It was found that participants generally supported the SPCH approach. Further, their discussions about palliative care needs and challenges in the WKB region and the SPCH have clearly assisted with refining the vision of this service model. Participants viewed the SPCH to be a practitioner/health service site(s)/network in a rural community designated to facilitate: two-way information exchange with the primary hub site; collaborative educational and training opportunities within the region and with the primary hub providers; information dissemination about throughout the region; enhanced service quality locally and in the region; raising the visibility and awareness of palliative care locally and in the region; and creating opportunities to support existing providers, to take measures against provider burnout, and to fill existing health human resources needs locally. The refined vision of the SPCH emerging from the case study interviews is summarized in Figure 2. Visually captured in this figure are the participants' service siting suggestions that the hub site: (1) need not be the community most closely situated to the primary hub location; (2) need not be the community most centrally located in the region; and (3) create linkages with other rural communities in order to further enhance palliative care provisioning throughout the region. Further related to the second purpose of this article, other service siting considerations raised by participants include: transportation networks; the locations of existing services; proximity to the hospital; existing connections or collaborations with the primary hub site; and the involvement of multiple stakeholders in the siting decision-making process.

Three consistent messages emerged about the SPCH across the four analytic themes described in detail above. First, there was a significant focus on the networking potential of the SPCH via establishing two-way information sharing channels being and the creation of educational and training opportunities. Such opportunities were thought to allow for working across care sites and professional groups, which were viewed as strengths of enhancing connections between sites providing specialized palliative care and smaller, more rural, communities. Second, participants' vision of a SPCH included further diffusing information and knowledge throughout the entire WKB region from the secondary hub, which is an important role for palliative care providers in the SPCH community to play. This would likely be done with ease given that many of the participants discussed regularly travelling to a number of communities in the region as part of their practice. This information and knowledge diffusion could happen in many forms, including by telemedicine, as frequently mentioned by participants, and/or emerging information sharing approaches such as the web-based e-hospice model proposed by Kuziemsky and colleagues [21]. Third, it is also clear from participants' discussion of this service approach that the SPCH need not be a physical location, such as an office in a clinic or hospital that could be used by specialists from primary hubs traveling to practice in rural communities or for on-site specialized teams, as this was not consistent with their vision. Rather, those connections they viewed as most desirable with the primary hub focused on networking. At the same time, they acknowledged that raising palliative care awareness through having dedicated enhancement of such services in a particular SPCH community via networking, and thus throughout the region, could ultimately lead to a hospice (i.e., a particular type of physical site) being located on-site, which was thought to be a highly desirable outcome.

Participants' focus on the networking potential of a SPCH in the interviews is consistent with recent calls to enhance the delivery of palliative care services in rural communities through the use of telemedicine and travelling clinics as a way to create innovative models of service delivery to meet demand [2,3], in that all of these approaches involve sharing expertise across communities. Importantly, the initial spatial analyses conducted in order to determine candidate SPCH communities [14,17] and the decision-making considerations raised in the findings shared above are extensible to any of these service approaches, among others. The SPCH approach cannot, however, be overly prescriptive or top-down as networking opportunities would need to focus on *local *palliative care service priorities, wherever the community of focus is located, in order to have the most impact and relevance. These priorities may include addressing identified barriers, such as a lack of round-the-clock care or local support for informal caregivers, and gaps, such as the lack of standardized palliative care data collection [17,22]. Such networking priorities would need to be established early on, while still visioning for a particular SPCH, so that they may be built into the roles and services.

It was noted at the outset that little research has been done to address the need to expand palliative care in rural Canadian communities in order to meet the anticipated service demand, particularly through the creation of innovative service models, with the exception of Kelley's [13] work. As a result of her long-term research regarding building community capacity for palliative care in rural Canada, Kelley [13] advocates for a thoughtful approach to enhancing service provision in rural communities. While the focus of the present study has been on location and decision-making factors as evidencing this desired thoughtfulness, Kelley's is on those elements that need to be present in rural communities in order to both initiate and sustain care provision. More specifically, she suggests that there are four phases of building local capacity for rural palliative care which, in general, are: (1) assessing that there is the necessary infrastructure and vision to support care provision; (2) having an event or person that stimulates change; (3) creating a dedicated palliative care team in the community; and (4) growing a program of palliative care. A key difference between the models is that in Kelley's approach an event or stimulus for change must come from the community itself or the community's response to the need for (or absence of) palliative care, while the SPCH approach may be implemented in response to numerous stimuli, including regional, provincial, or even national-level decision-makers' desires to enhance rural palliative care. The community capacity building model is, however, not inconsistent with the SPCH approach discussed herein. There is recognition in both that an assessment of local service provision, strengths, and challenges must take place before any enhancement is to be pursued. Networking among care providers is also an important feature of both approaches. These points of commonality between the models provide some direction as to what should be considered when developing any approach to address the palliative care need in rural communities that also meets the unique and particular circumstances posed by enhancing service provision in such places.

### Limitations

A potential limitation is that we present the findings of a single case study in this article. While this is consistent with the case study methodology, whereby depth is sought and a full understanding of the relevant context is crucial [23], having a single case study limits the ability compare the findings emerging from the WKB to those from other rural regions. As such, it is not possible to generalize the findings. However, as generalizability is not a goal of qualitative research [24] we do not believe that this serves as a true limitation, nor does it limit the quality of the findings.

A second potential limitation is that most participants responded to the Likert scale questions using 'very important' or 'critically important' and as such there is little differentiation in the relative importance of the factors probed, thus rendering this data to be of little use. In future SPCH research the inclusion of such questions needs to be reconsidered, including the wording of the questions and the factors being probed. In acknowledging this limitation, we have only used this data as secondary in order to support findings emerging from the interviews.

A third potential limitation is that the use of the examples of videoconferencing and mentorship relationships in the explanation of the hub approach given during the interviews may have increased the likelihood of participants raising these features in their discussions of the SPCH approach. To minimize this we were careful to explain that the examples being offered were intended only to ensure that participants had a common and minimal understanding of the issue of focus. It is clear from the findings shared above that the participants did not particularly champion these examples as becoming features of a SPCH, instead more commonly mentioning teleconferencing and tele-medicine over videoconferencing and education and training opportunities over mentorship. The findings, thus, do not indicate that our inclusion of these examples limited participants' interpretation of the potential of the SPCH.

### Importance

It was mentioned at the outset of this article that because of population aging there is an impending and essential need to address issues of inadequate supply of palliative care in many developed nations, including in rural areas. This article has focused on the case of a rural region of BC, Canada; however, this health service issue affects a number of other Canadian provinces and countries [2,3,12], thus making the SPCH approach and the findings shared herein to be of use beyond the case study region. A particularly useful parallel may be drawn between the context of the WKB region and that found in rural Australia, where issues of distance and isolation are clearly comparable. These two countries both face an increasing demand for palliative care, a greying or aging of the population, a rise in aging-in-place and the movement of retirees from cities into the countryside, and a need to enhance service provision in rural areas [2,6-8,16,25]; thus, the findings presented here are likely to be highly relevant to the Australian case. This is but one example of another jurisdiction that shares similar demands and context and thus may find use in considering the implications of the consistent messages of the present study, the points of commonality identified with the Kelley [13] model, and the SPCH approach more generally, among other things, for its own situation.

### Future Research

There are many further considerations for the SPCH approach that have not been addressed in the present study that require additional research. Economic analyses are certainly needed in order to evaluate cost and feasibility. Additional case studies also need to be undertaken in different decision-making and service provision environments in order to determine how unique or common the vision put forth by formal and informal palliative care providers in the WKB region is to those in rural communities with varied circumstances. Finally, further research attention must be given to rural communities' concerns regarding service centralization, in that while the intent of the SPCH approach is to diffuse palliative care expertise from existing primary hubs where services and knowledge are already centralized outwardly to rural communities, some participants were concerned that the secondary hub would further centralize things, just at a more local scale.

Our own most immediate next steps will be to complete the analysis and write-up of all five main analytic issues identified in the qualitative dataset, as reported in the methods section. Following this we aim to revisit our original siting model created before the interviews were completed [17] and to use the qualitative dataset to revise it by inserting some of the factors identified in the present analysis. We then aim to move ahead with running the revised siting model in other Canadian provinces and to use the results to identify additional case study communities in which to conduct interviews. Additional case study interviews will either confirm the vision of the SPCH put forth by formal and informal palliative care providers in the WKB region, or further refine the overall vision of the SPCH approach by identifying new features to consider and/or service siting factors.

## Conclusion

Palliative care tends to slip under the radar of public and policy attention, and this is especially so in rural and small town settings where the focus of attention is typically on securing and retaining emergency and acute care resources and personnel. The idea of a SPCH provides a point of focus for rural health care providers, policy makers, and the wider public to make concerted local and regional efforts to enhance the bundling and building of palliative care resources along with service provision. As the participants noted, the hub also has the potential to raise awareness of palliative care issues, facilitate access to more specialized services, and allow for greater access to training and education opportunities in challenging care settings. Finally, a hub can serve as a platform for the exchange of knowledge and expertise within and beyond traditionally less well-connected rural centres.

The visioning promoted herein combines elements of rational planning criteria with social networking principles to facilitate the exchange of ideas and expertise. Importantly, there is no 'cookie-cutter' approach to palliative care service planning, whether in urban or rural settings. Bringing a variety of perspectives together (e.g., different health and social care sectors, expert and lay opinion) maximizes local input about client needs and preferences, helps to identify gaps in local services, and may facilitate greater opportunities for local collaboration and the coordination of efforts that strengthen local systems of support. A more consultative approach also promises to avoid the pitfalls of being perceived to be top-down by establishing a middle ground for ideas and innovations to come forward from local and non-local sources. This is useful not only to resolve present palliative care service concerns, but also to enable knowledge transfer and capacity building in rural settings. In these ways, a visioning exercise, such as that presented herein, offers a greater likelihood for participants to take ownership of the SPCH idea, particularly as participants are presented with opportunities to shape the form and content of the hub.

## Abbreviations

BC: British Columbia; SPCH: Secondary Palliative Care Service Hub; WKB: West Kootenay-Boundary Region

## Competing interests

The authors declare that they have no competing interests.

## Authors' contributions

VAC led the development and writing of this manuscript. She co-leads the study and was integrally involved in all steps of qualitative data collection and analysis. HC contributed substantially to the data analysis and to writing this manuscript. She also conducted the interviews reported on herein. NS co-leads the study and has thus been centrally involved in all stages of development and design. She edited the manuscript and created all figures. NH has contributed to interpreting the findings and also drafted the conclusion section of this manuscript. All authors have read and approved the final manuscript.

## Pre-publication history

The pre-publication history for this paper can be accessed here:



## Supplementary Material

Additional file 1 **Supplemental Table S1**. This file consists of a table that displays the Likert questions and their responses. Click here for file
