# Supplementary material for: Visioning for secondary palliative care service hubs in rural communities: a qualitative case study from British Columbia's interior
Source: BMC Palliat Care. 2009 Oct 9;8:15. doi: 10.1186/1472-684X-8-15 (PMC2763848; doi:10.1186/1472-684X-8-15)
Supplement: Additional file 1 — Supplemental Table S1. This file consists of a table that displays the Likert questions and their responses. [file 1472-684X-8-15-S1.DOC]

| **#** | **Question** | *Total # of responses** | Not at all important | Somewhat important | Fairly important | Very important | Critically important |
| --- | --- | --- | --- | --- | --- | --- | --- |
| 1 | Having a free-standing palliative care facility in my community is | *30* | 0 | 3 | 2 | 9 | 16 |
| 2 | Having a family physician in my community is | *30* | 0 | 1 | 0 | 2 | 27 |
| 3 | Having a family physician who has experience providing palliative care in my community is | *30* | 0 | 0 | 1 | 8 | 21 |
| 4 | Having home care available to people needing palliative care in my community is | *30* | 0 | 0 | 1 | 1 | 28 |
| 5 | Having home care providers based in my community is | *30* | 1 | 0 | 1 | 3 | 25 |
| 6 | Having a nurse with palliative care specialized training in my community is | *30* | 0 | 0 | 1 | 7 | 21 |
| 7 | Having a nurse practitioner with palliative care specialized training who can diagnose and prescribe in my community is | *29* | 0 | 1 | 4 | 10 | 15 |
| 8 | Having a car in my community is | *30* | 0 | 2 | 4 | 11 | 13 |
| 9 | When driving within my community, weather conditions are | *30* | 2 | 0 | 5 | 13 | 10 |
| 10 | Weather conditions in my region, when travelling outside my community are | *30* | 0 | 0 | 2 | 9 | 19 |
| 11a | Receiving care as fast as possible is | *30* | 0 | 0 | 2 | 10 | 18 |
| 11b | Receiving palliative care as fast as possible is | *21* | 0 | 0 | 3 | 7 | 11 |
| 12a | Receiving the best quality care possible is | *30* | 0 | 0 | 0 | 8 | 22 |
| 12b | Receiving the best quality palliative care possible is | *21* | 0 | 0 | 1 | 6 | 14 |
| 13a | Receiving the most technologically advanced care as possible is | *29* | 0 | 1 | 3 | 17 | 8 |
| 13b | Receiving the most technologically advanced palliative care as possible is | *21* | 1 | 2 | 3 | 10 | 5 |
| 14 | The freedom to die at home or at your chosen location is | *30* | 0 | 0 | 1 | 9 | 20 |
| 15 | If your desire is to die at home and you cannot, how important is it to die in your own community | *30* | 0 | 1 | 2 | 6 | 21 |
| 16 | If your desire is to die in your own community and you cannot, how important is it to die in your own region | *30* | 1 | 0 | 5 | 8 | 16 |
| 17 | Considering the (burden of) cost of caring for a dying family member is | *28* | 1 | 0 | 5 | 8 | 14 |
| 18 | Supporting people in the community who are caring for a dying loved one is | *30* | 0 | 0 | 1 | 9 | 20 |
| 19 | Providing people with information about local palliative care resources is | *30* | 0 | 0 | 1 | 13 | 16 |
| 20 | Training service providers in rural palliative care is | *30* | 0 | 0 | 0 | 11 | 19 |
| 21 | Providing educational opportunities for rural palliative care providers in their own communities is | *30* | 0 | 0 | 5 | 10 | 15 |

* some participants chose not to respond to particular questions
